# Local Field Potential Biomarkers of Non‐Motor Symptoms in Parkinson's Disease: Insights From the Subthalamic Nucleus in Deep Brain Stimulation

**DOI:** 10.1111/ejn.70046

**Published:** 2025-03-04

**Authors:** Marc‐Antoine Gobeil, Albert Guillemette, Meziane Silhadi, Laurence Charbonneau, David Bergeron, Adan‐Ulises Dominguez‐Vargas, Numa Dancause, Nicolas Jodoin, Elie Bou Assi, Florin Amzica, Sami Obaid, Marie‐Pierre Fournier‐Gosselin

**Affiliations:** ^1^ Department of Medicine University of Montreal Montreal Quebec Canada; ^2^ Centre de Recherche du Centre Hospitalier de l'Université de Montréal (CRCHUM) Montreal Quebec Canada; ^3^ Department of Surgery University of Montreal Montreal Quebec Canada; ^4^ Department of Neurosciences University of Montreal Montreal Quebec Canada; ^5^ Centre Interdisciplinaire de Recherche sur le Cerveau et l'Apprentissage (CIRCA) Montreal Quebec Canada; ^6^ Division of Neurology Centre Hospitalier de l'Université de Montréal (CHUM) Montreal Quebec Canada; ^7^ Department of Stomatology University of Montreal Montreal Quebec Canada; ^8^ Service of Neurosurgery, Centre Hospitalier de l'Université de Montréal (CHUM) Montreal Quebec Canada

**Keywords:** deep brain stimulation, local field potentials, non‐motor symptoms, Parkinson's disease, subthalamic nucleus

## Abstract

Non‐motor symptoms can severely affect the quality of life of Parkinson's disease‐afflicted patients, with the most common ones being pain, sleep impairments, and neuropsychiatric manifestations. In advanced cases, complex fluctuations of motor and non‐motor symptoms can occur despite optimal medication. Research on deep brain stimulation of the subthalamic nucleus suggests that it may provide benefits for treating non‐motor symptoms in addition to improving motor symptoms. With recent advancements in deep brain stimulation technology, simultaneous recording of local field potentials and delivery of therapeutic stimulation is possible. This opens new possibilities for better understanding the pathophysiology of non‐motor symptoms in Parkinson's disease and for identifying potential electrophysiological biomarkers that accurately represent these symptoms. Specifically, this review aims to highlight potential local field potential biomarkers of non‐motor symptoms in the subthalamic nucleus. The main findings indicate that activities in the beta frequency band are associated with nociception and sleep impairments such as insomnia and rapid eye movement sleep behavior disorders. Additionally, activities in the theta and alpha frequency bands seem to reflect neurocognitive manifestations, including depression and impulse control disorders. A better understanding of these biomarkers could improve the clinical management of non‐motor symptoms in Parkinson's disease. They hold promise for adjusting deep brain stimulation parameters in open‐loop settings and might eventually be applied in closed‐loop deep brain stimulation systems, though their true impact remains uncertain.

## Graphical abstract

This review highlights potential electrophysiological biomarkers within the subthalamic nucleus representing non‐motor symptoms in Parkinson's disease. Findings suggest that pain and sleep disturbances may correlate with beta band activity, while neuropsychiatric symptoms may be associated with the alpha and theta bands. These biomarkers can increase our understanding of the mechanisms involved in these non‐motor symptoms and eventually be used to adjust DBS therapy in clinical settings.

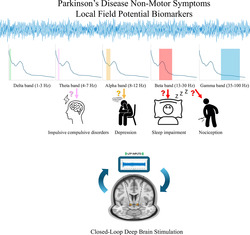

**Abbreviations**
ACC: anterior cingulate cortex
ANN: artificial neural networks
DBS: deep brain stimulation
EDS: excessive daytime sleepiness
LFP: local field potentials
MeSH: Medical Subject Headings
NREM: non‐REM
PD: Parkinson's disease
PRISMA: Preferred Reporting Items for Systematic Reviews and Meta‐Analyses
RBD: REM behavior disorder
REM: rapid eye movement
SANRA: Scale for the Assessment of Narrative Review Articles
STN: subthalamic nucleus
SVM: support vector machine

## 1 Introduction

Parkinson's disease (PD) is a neurodegenerative disorder that affects more than 10 million people worldwide (de Lau and Breteler 2006; Flouty et al. 2022). PD is characterized by four cardinal motor features: tremor at rest, rigidity, akinesia (or bradykinesia), and postural instability (Jankovic 2008). Most of the existing literature on PD focuses on motor symptoms. However, non‐motor symptoms are also prevalent among PD patients, with nearly all experiencing at least one non‐motor disturbance (Kim et al. 2013; Krishnan et al. 2011). These symptoms include pain, sleep impairments and neuropsychiatric manifestations. Their pathophysiology remains to be fully elucidated, but studies suggest a complex interaction involving dysfunction in both dopaminergic and non‐dopaminergic systems (Barone et al. 2009). Non‐motor symptoms often appear in the early stages of the disease, contributing to severe disability, impaired quality of life, and shortened life expectancy (Chaudhuri et al. 2006). Thus, non‐motor symptoms play a significant role and should not be overlooked in the management of PD (Foltynie et al. 2024).

The first line of treatment for PD is pharmacological, with levodopa being the gold standard therapy (Hauser 2009). Nevertheless, for advanced cases of PD with major motor symptom fluctuations, deep brain stimulation (DBS) can be considered, as it improves off‐period akinesia and on‐period dyskinesia (Krack et al. 2003). DBS entails the stereotactic implantation of electrodes in specific anatomical sites, such as the subthalamic nucleus (STN), a canonical PD target (Fox et al. 2011). STN‐DBS has been well established in improving motor symptoms in PD (Cury et al. 2018; Kalia et al. 2013; Schuepbach et al. 2013), and emerging research also suggests potential benefits for alleviating non‐motor symptoms (Baumann‐Vogel et al. 2017; Cicolin et al. 2004; Kim and Jeon 2021).

Recent advancements in neurostimulation technology have led to the development of devices capable of simultaneously recording local field potentials (LFP) and delivering therapeutic stimulation (Thenaisie et al. 2021). LFP represents the low‐frequency components of extracellular electrical potentials, reflecting the dynamics and synchronized input activity of nearby neuronal and glial units (Belasen et al. 2016; Maling and McIntyre 2016).

**FIGURE 1:**
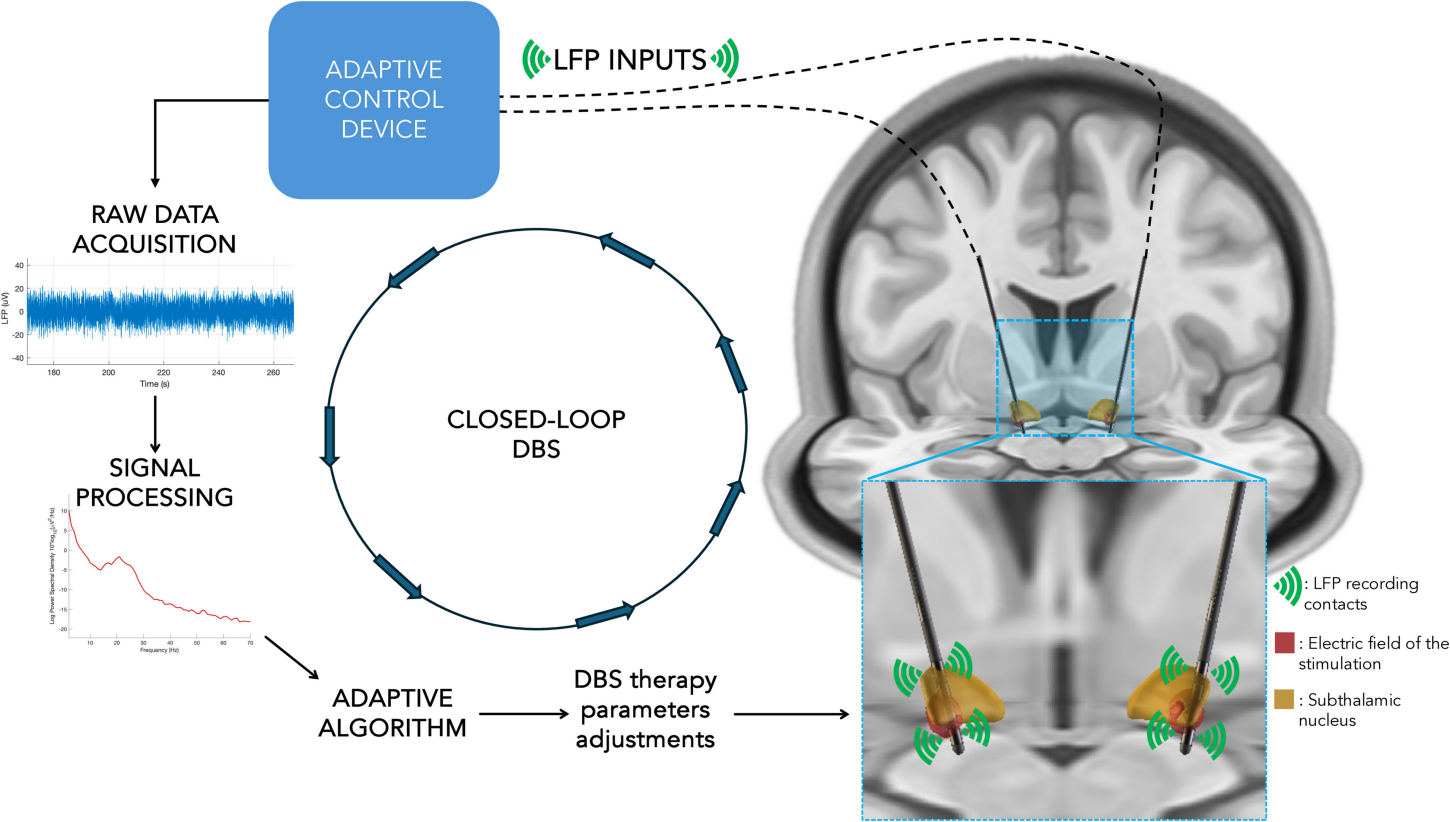
Theoretical model of a closed‐loop deep brain stimulation (DBS) system for the treatment of Parkinson's disease represented in a Montreal Neurological Institute (MNI) template MRI space. Local field potentials (LFP) data processing and adaptive algorithm adjustment of DBS parameters (Ewert et al. 2018; Fonov et al. 2011; Neudorfer et al. 2023).

Some LFP features correlate with the severity of motor symptoms (such as power in the beta band in the STN [Kühn et al. 2008] or subharmonic 1:2 entrainment of the STN stimulation in the neocortex [Oehrn et al. 2024]), potentially allowing for the modulation of stimulation intensity based on the continuous measurement of an electrophysiological biomarker (“adaptative” or “closed‐loop” DBS) (Neumann et al. 2023). This holds promise to improve the clinical efficacy of STN DBS (Oehrn et al. 2024) (see Figure 1). In addition, STN LFP recordings from sensing‐enabled DBS systems offer a unique window into the electrophysiology of the STN in various physiological and pathological states, as well as its association with various non‐motor symptoms. The STN receives inputs from many non‐motor frontal‐subcortical pathways involved in cognition and emotional regulation (Cummings 1993); hence, the STN electrophysiology may reflect fluctuations in various non‐motor states. In this manuscript, we review the spectral representation of LFP in the STN of patients with PD in relation to common non‐motor symptoms such as pain, sleep disturbance, depression and other neuropsychiatric manifestations.

## 2 Methods

The objective of this narrative review was to identify changes in LFP spectral representation in relation to common non‐motor symptoms of PD, such as pain, sleep disturbance, depression and neuropsychiatric manifestations. Physiological processes like sleep or sensory processing were also assessed. Our search focused on studies of patients with PD who were implanted with a sensing‐enabled DBS device in the STN. This narrative review was conducted following best practices for narrative synthesis. To ensure methodological rigor, we used the Scale for the Assessment of Narrative Review Articles (SANRA) as a quality assessment tool (see Supporting Information) (Baethge et al. 2019). Additionally, we adapted relevant components of the Preferred Reporting Items for Systematic Reviews and Meta‐Analyses (PRISMA) guidelines to enhance transparency in reporting (see Supporting Information) (Page et al. 2021). All experimental studies published in English up to 2024 were included. A comprehensive search was performed using MEDLINE, applying a search strategy that combined both medical subject headings (MeSH) and keywords. We used the following search strategy: “Parkinson's Disease” AND “Subthalamic Nucleus” AND “Local Field Potentials” AND [“Non‐Motor Symptoms” OR “Pain” OR “Nociception” OR “Sleep” OR “Insomnia” OR “Excessive Daytime Sleepiness” OR “Neuropsychiatric” OR “Cognitive” OR “Behavior” OR “Depression” OR “Impulse Control Disorder”]. We did not comprehensively review animal studies reporting LFP data, as well as studies reporting LFP from other canonical DBS targets (e.g., thalamus, globus pallidus internus, and pedunculopontine nucleus) for the purpose of this work, although we did consider this broader literature to discuss and interpret our results. Two reviewers (M.A.G. and A.G.) independently screened and assessed each article to assess their relevance, and disagreements were solved through discussions. Although the definition of frequency bands has slight variations across studies, we used the following frequency ranges in this manuscript: delta (1–3 Hz), theta (4–7 Hz), alpha (8–12 Hz), beta (13–30 Hz) and gamma (35–100 Hz) (Ricciardi et al. 2023).

## 3 Results

### 3.1 Pain and Nociception

In PD, pain is one of the most common non‐motor symptoms and is a significant source of suffering and disability (Flouty et al. 2022; Politis et al. 2010). Pain onset can precede motor symptoms by several years (Ainhi and Ha 2012; Young Blood et al. 2016). Its overall prevalence in PD is between 60% and 85% (Beiske et al. 2009; Fil et al. 2013; Lee et al. 2006; Parker et al. 2020; Rana et al. 2013; Valkovic et al. 2015). Therefore, research on PD‐related pain's electrophysiological representation in the STN is important to allow better understanding and management of this non‐motor symptom (Bouthour et al. 2019; Ricciardi et al. 2023; van Wijk et al. 2023).

PD‐related pain etiology is multifactorial (Ford 2010), and the most common type in PD is nociceptive musculoskeletal pain (Gierthmuhlen et al. 2010; Skogar and Lokk 2016). Pain can be divided into two main classes: sensory‐discriminative pain and affective‐motivational pain (Belasen et al. 2016; Treede et al. 1999). On the one hand, the sensory‐discriminative component of pain is mediated by a lateral pathway, which includes lateral thalamic nuclei and primary as well as secondary somatosensory cortices (Mostofi et al. 2021). Additionally, the dorsal fundus of the posterior insula serves as an important relay station in ascending pain pathways, transmitting noxious thermal and mechanical painful stimuli information (Bergeron et al. 2021). Exacerbation of sensory‐discriminative pain is mainly caused by a sensitization to noxious stimuli, which is reflected by a reduction of pain thresholds in parkinsonian patients (Djaldetti et al. 2004). As shown in an extensive systematic review and meta‐analysis, PD patients have lower thermal and mechanical pain thresholds than healthy subjects during quantitative sensory testing (Sung et al. 2018). This phenomenon can be explained by central pain processing alterations, mainly caused by dopaminergic deficiency in the nigrostriatal and mesolimbic pathways, leading to aberrant function of cortico‐basal ganglia loops responsible for pain modulation (Alberico et al. 2015; Granovsky et al. 2013). Indeed, dopaminergic projections from the ventral tegmental area to the nucleus accumbens, prefrontal cortex and cingulate cortex are known to have a role in central mechanisms of analgesia, which explains how dopaminergic deficiency can exacerbate pain symptoms (Mostofi et al. 2021).

On the other hand, the affective‐motivational component of pain is mediated through a medial pathway, including the periaqueductal gray matter, medial thalamic nuclei, anterior cingulate cortex (ACC) and anterior insula (De Ridder et al. 2022). Notably, the anterior insula projects to various limbic structures involved in attributing emotional valence to pain, such as the ACC. The ACC is responsible for affective‐motivational, cognitive, evaluative and memory aspects of pain (Vogt and Sikes 2000). In functional studies, it is reported that patients with PD‐related pain have a higher activation in their ACC compared to PD patients without pain manifestations (Brefel‐Courbon et al. 2013). Furthermore, STN‐DBS could modulate neuronal firing in the ACC (Belasen et al. 2016), suggesting dopamine deficiency might also be implied in the emotional exacerbation of pain in PD.

In a meta‐analysis and systematic review evaluating the influence of continuous STN‐DBS and globus pallidus internus DBS on chronic pain in PD, Flouty et al. (2022) identified a significant improvement of 40% in pain scores (Flouty et al. 2022). Interestingly, STN‐DBS can increase pain thresholds and reduce sensitivity to painful stimuli in PD‐related pain patients (Belasen et al. 2017; de Andrade et al. 2012; Dellapina et al. 2012). The exact process explaining how STN‐DBS achieves pain reduction remains unclear. Part of this therapeutic effect may be explained by the reduction of motor symptoms (Garcia‐Garcia et al. 2016). Indeed, alleviating the burden of motor symptoms in PD may reduce the psychological and emotional distress of patients, thereby reducing their subjective painful experience. On the other hand, STN‐DBS may also exert its effect by modulating central pain processing structures in the brain, notably through stimulation of limbic components of the STN influencing the nucleus accumbens (Le Jeune et al. 2010), through modulation of the ACC or through modulation of other central structures involved in the pain pathways mentioned earlier (Belasen et al. 2016; Flouty et al. 2022).

Some authors have reported changes in STN LFP power spectrum with various sensory stimuli (Belasen et al. 2016; Parker et al. 2020). For instance, Belasen et al. (2016) conducted a study on the influence of mechanical and thermal painful stimuli on single unit activity and LFP activity in the STN. Single unit activity increased in response to noxious mechanical, noxious pressure, and noxious thermal stimuli. LFP analyses revealed increased alpha activity in response to non‐painful mechanical stimuli. No significant oscillatory change in STN LFP was observed after thermal stimuli. This study was the first to demonstrate that mechanical and thermal stimuli alter oscillations in the basal ganglia of PD patients (Belasen et al. 2016). In another study, Parker et al. (2020) investigated LFP changes in the STN in response to mechanical pain stimulation. A significant power increase in the beta frequency band between mechanical painful and non‐painful stimuli was observed. They also noted a significant decrease in pain‐induced beta activity during active DBS stimulation compared to when stimulation is not delivered. This could be explained by excessive synchronization of pain‐related low‐beta frequency neurons in the STN in PD‐related pain states. Thus, DBS could attenuate these symptoms through neuronal desynchronization (Parker et al. 2020). Interestingly, excessive beta band neuronal synchronization in the STN is also associated with bradykinesia and rigidity in PD (Kuhn et al. 2004; Little and Brown 2012; Ray et al. 2008). Some of the results reported in the literature are also inconsistent. For instance, Belasen et al. (2016) have not found a significant increase of LFP beta power in the STN after mechanical noxious stimuli (Belasen et al. 2016), whereas Parker et al. (2020) did (Parker et al. 2020). To date, no data is available on the correlation of STN LFP with the spontaneous fluctuation of subjective pain intensity in parkinsonian patients who experience chronic pain. We cannot infer that LFP power spectrum changes observed after acute stimuli will help predict fluctuations in subjective chronic pain intensity, as these represent distinct physiological mechanisms and involve distinct neuronal pathways. In addition, there are no data suggesting that DBS parameter changes can selectively improve the control of chronic pain, independently of motor symptoms. Therefore, more research is needed before we can envision adding electrophysiological markers of pain to multi‐input closed‐loop DBS systems. Nevertheless, STN LFP analysis could serve as an additional tool to enhance our understanding of the various subtypes of chronic pain (e.g., low‐back pain, neuropathic burning‐like pain, or rigidity associated‐pain) experienced by patients with PD.

### 3.2 Sleep

Sleep–wake disturbances such as insomnia, rapid eye movement (REM) sleep behavior disorder (RBD), and excessive daytime sleepiness (EDS) are commonly associated with PD. In a meta‐analysis performed by Maggi et al. (2023), the pooled prevalence of insomnia, RBD, and EDS was 44%, 46%, and 35%, respectively (Maggi et al. 2023). Considering the important negative impact of these disturbances on the quality of life of patients with PD (Neikrug Ariel et al. 2013; Gómez‐Esteban et al. 2011), adequate treatment of these symptoms is paramount. The physiological mechanisms involved in the sleep–wake cycle regulation are complex. Studies show that dopamine from the substantia nigra, with its effects on striatal neurons, holds an important role in the regulation of sleep, particularly in the REM stage (Lima et al. 2007; Qiu et al. 2016). Through a thalamo‐cortical‐basal ganglia oscillatory network, the basal ganglia, with its outputs to the intralaminar and reticular nuclei of the thalamus, brainstem, and cerebral cortex, play a crucial role in regulating the sleep–wake cycle in both slow‐wave and REM sleep (Hasegawa et al. 2020).

Sensing‐enabled DBS systems provide an interesting opportunity to monitor physiological processes and sleep disturbances in patients with PD. The first study to use STN LFP to assess the sleep of PD patients was conducted by Urrestarazu et al. (2009) on 10 participants 2–4 days after their DBS surgery. LFP recordings were performed before the internalization of the DBS system, through the external connection of the implanted macroelectrodes. During stage two (S2) of non‐REM (NREM) sleep, they reported an increase in power in delta, theta and alpha bands in comparison to wakefulness. In S2 and stage four (S4), beta power was significantly lower than during wakefulness. In REM sleep, beta power was slightly higher than during wakefulness and clearly higher than in S2 and S4. The power increase in REM sleep affected almost exclusively the high‐beta range (20–30 Hz), leaving the low‐beta range (13–20 Hz) lower than during wakefulness (Urrestarazu et al. 2009). In subsequent studies, these changes in frequency band power during NREM sleep stages (N1, N2, and N3) were also observed. Specifically, power increased in the delta, theta, and alpha bands, while it decreased in the beta and gamma bands (Balachandar et al. 2024; Thompson et al. 2018; van Rheede et al. 2022). These changes were particularly noted in the N2 and N3 stages when compared to wakefulness (Anjum et al. 2024; Chen et al. 2019). Interestingly, STN DBS is known to reduce beta power during NREM sleep, perhaps to more physiologic levels (Anjum et al. 2024), although the “normal range” of STN beta power has never been characterized in patients without PD. In a therapeutic perspective, Yin et al. (2023) estimated that using the same beta power thresholds during wakefulness and sleep as input for closed‐loop DBS would lead to understimulation and undertreatment of beta alterations during NREM sleep (Yin et al. 2023).

During REM sleep, a significant decrease in delta, theta and alpha bands was noticed, while beta and gamma bands returned to levels comparable to wakefulness (Chen et al. 2019). Notably, beta power was elevated during REM sleep without atonia (Yin et al. 2024). Additionally, this elevation in beta power during REM sleep was positively correlated with the degree of atonia loss. This beta power increase occurred approximately 200 ms before the activation of chin electromyogram activities. This association between beta power and chin muscle activity during REM sleep was correlated with the clinical severity of REM sleep disorder (Yin et al. 2024). Interestingly, it was also observed that beta power increased just before sleep interruption. This specific indicator of sleep interruption, in the beta band, could act as a therapeutic target used to manage sleep disorders (Anjum et al. 2024). These studies also reported significant interindividual variability in the relative power of each frequency band during different sleep stages. This suggests that an individualized approach, tailored to each patient, should be considered to develop accurate predictive models. To address this, support vector machine (SVM) classifier models, feed‐forward artificial neural networks (ANN) and random forest classifiers were used (Anjum et al. 2024; Balachandar et al. 2024; Chen et al. 2019; Christensen et al. 2019; Thompson et al. 2018). Most of these models employed standard 30‐s LFP recording windows and/or shorter 5‐s recording windows for training and testing. For larger recording windows, the accuracy of the models surpassed 90% in most instances (Anjum et al. 2024; Christensen et al. 2019; Thompson et al. 2018), whereas for shorter temporal windows, accuracy seemed to be lower (Anjum et al. 2024; Chen et al. 2019).

To date, there is limited data suggesting that adjusting DBS parameters to sleep cycles is beneficial in patients with PD. Overall, continuous DBS of the STN is known to increase sleep quality in patients with PD (Zhu et al. 2023). In a recent closed‐loop DBS for motor symptoms trial (using subharmonic 1:2 entrainment of STN stimulation in the subdural cortical electrode as a biomarker of motor state), the majority of the night was spent at high stimulation amplitude (due to the decreased STN‐cortical coupling during sleep). This led to an increase in electrical power consumption. Additionally, sleep quality was not significantly improved in closed‐loop versus continuous DBS (Oehrn et al. 2024). Some authors have highlighted that using beta fluctuations as the input biomarker in closed‐loop STN DBS may lead to unwanted reductions in stimulation amplitude during NREM sleep (which is associated with lower beta), inadvertently leading to increased sleep disturbances (Yin et al. 2023). In a small pilot study, Gilron et al. (2021) developed a dual algorithm design with two independent detectors, one used to track sleep state (wake/sleep) and the other used to track parkinsonian motor state (medication‐induced fluctuations). The algorithm could successfully detect the transition to sleep (using alpha and theta power changes), during which it switched to a continuous DBS mode, then switched back to a closed‐loop mode (using beta power changes) during wakefulness. This trial was designed to assess the feasibility of this approach, rather than its impact on sleep quality (Gilron et al. 2021).

Altogether, across studies, there is a consistent observation of increased power in lower frequency bands (delta, theta, alpha) during NREM sleep and decreased beta power compared to wakefulness. REM sleep often shows distinctive patterns, including higher beta power than during NREM. Various machine learning models, including SVM and ANN, have been developed to predict sleep stages based on LFP data. These models have achieved high accuracy and could potentially be integrated to modulate the stimulation based on sleep stages, as well as interrupting neuronal activity linked to sleep impairments. More data is needed on the effect of DBS parameter changes on sleep impairment in the different sleep stages before these sleep decoding algorithms can be meaningfully integrated in multi‐input closed‐loop DBS algorithms.

### 3.3 Neuropsychiatric Manifestations

Cognitive impairment and neuropsychiatric symptoms such as altered mood, depression, and impulse control disorders are common non‐motor symptoms of PD (Balestrino and Martinez‐Martin 2017). The prevalence of major depressive disorder among PD patients was 17%, while 22% are affected by minor depression, and 13% experience dysthymia (Reijnders et al. 2008). In addition, for impulse control disorders, which are consequences of dopaminergic replacement therapy, the estimated prevalence ranges between 3% and 4% (Zhang et al. 2014). The risk of impulse control disorders is increased in the initial adjustment phase to STN DBS, as the requirements for dopaminergic medication are reduced by the stimulation; hence, patients may experience a transient excess in dopamine during stimulation and medication adjustments (Merola et al. 2017). The specific mechanisms underlying these non‐motor symptoms are still largely unknown, which explains the limited development of effective therapies. Therefore, intracerebral recordings with DBS offer an opportunity to understand the mechanisms underlying these symptoms at the neuronal and physiological levels, potentially leading to improved treatments.

An extensive review of the spectral representation of neuropsychiatric and cognitive impairments in PD was performed by Ricciardi et al. (2023). Through a framework based on symptoms and behaviors, these authors assessed: (1) affect and emotional processing, (2) executive control, (3) subjective valuation (reward and cost evaluation), (4) motor control, and (5) learning and outcome‐related updating (Ricciardi et al. 2023). A summary of their findings, along with results from other key articles, is provided in the following sections.

#### 3.3.1 Affect and Emotional Processing

Alpha band activity within the STN has been associated with the occurrence of depressive symptoms, their severity, and the processing of emotional stimuli (Ricciardi et al. 2023). For instance, a retrospective case–control study of PD patients with and without depressive symptoms showed a resting‐state increase in alpha power and a decrease in theta power in those with depressive symptoms. This increase in alpha power was positively correlated with the severity of depressive symptoms, while theta power was negatively correlated (Sun et al. 2021). Furthermore, Huebl et al. (2011) reported reduced alpha STN power in reaction to pleasant stimuli in participants with mild to moderate depressive symptoms. Additionally, when presented with unpleasant stimuli, patients with depression had a larger decrease in alpha power, which was significantly correlated with the Beck depression inventory after 3 months of DBS therapy (Huebl et al. 2011). Alpha power may serve as an indicator of depressive symptoms severity and could potentially be used for managing depressive symptoms in PD.

#### 3.3.2 Executive Control, Impulsivity, and Decision‐Making

Regarding executive control in PD patients with impulse control disorders, a literature review conducted by Ricciardi et al. (2023) suggests that low‐frequency neuronal activity in the theta range might be associated with impulsive‐compulsive behaviors (Ricciardi et al. 2023). Notably, it was reported that for pathological gamblers, low‐frequency synchronization was significantly greater during conflictual economics decisions than during non‐conflictual economics decisions (Rosa et al. 2013). Regarding high‐order executive control and reflection impulsivity (i.e., the tendency to gather and evaluate information before making a decision [Kagan 1966]) of PD patients without impulse control disorders, they noted that theta band activity increases when faced with an incongruent situation involving conflict processing (Ricciardi et al. 2023). This process is likely facilitated by interactions with other frequency bands, including those within the motor cortex‐subthalamic beta network (Herz et al. 2017). Furthermore, a link between a component of prefrontal cortex theta activity and its synchronization with the STN during conflict detection is reported (Herz et al. 2016; Zavala et al. 2016, 2018). Finally, for subjective valuation in terms of reward/cost evaluation and choice impulsivity, it is suggested that the STN plays a crucial role in evaluating reward, risk, and effort. These processes also appear to be associated with low‐frequency activity, particularly in the theta range (Ricciardi et al. 2023). For example, Pearson et al. (2017) reported a theta band power increase preceding motor action during the decision period of a risk‐taking paradigm (Pearson et al. 2017). Overall, this suggests that impulsivity, high executive control, and reward/cost evaluation are represented through low‐frequency components, mostly in the theta band. Monitoring and modulating the theta band in the medication adjustment phase in patients who underwent STN DBS may help the timely detection of impulse control disorders before their full clinical expression and consequences.

#### 3.3.3 Motor Inhibition

The beta band shows the strongest task‐modulated LFP signal, decreasing just prior to and during movement, and increasing after movement termination. This phenomenon is commonly referred to as beta event‐related desynchronization and synchronization, respectively (Herz et al. 2024). Conversely, motor inhibition is associated with an increase in beta band power (Ricciardi et al. 2023). Additionally, during a response inhibition task (i.e., modified Stop Signal Task), Benis et al. (2014) reported that beta band activity was higher in the STN when participants had to inhibit a response. This electrophysiological response was predictive of the subjects' inhibitory performances during the task (Benis et al. 2014). Interestingly, Alegre et al. (2013) also reported a bilateral decrease in gamma STN power and in cortico‐subthalamic coherence when patients successfully inhibited their response during the “ON” medication state. This gamma band power reduction was not observed in their four PD participants with impulse control disorders. This suggests that motor inhibition could be mediated by a gamma band power reduction (Alegre et al. 2013).

#### 3.3.4 Reward Learning and Updating

For reward learning and updating, Schroll et al. (2018) recorded LFP during a reinforcement‐learning paradigm. During feedback presentation (i.e., information about the success or failure of a response), these authors reported that beta band activity was positively correlated with the magnitude of the reinforcement. However, when responding, alpha and low beta band activity were negatively correlated with previous reinforcement magnitudes, meaning that the stronger the previous reinforcement, the weaker the power of these oscillations during the next response. They did not identify any changes in beta activity caused by reinforcement prediction errors (i.e. difference between the expected reward and the actual reward) or caused by patients' tendencies to either repeat or adapt their responses (Schroll et al. 2018).

In summary, current research has identified biomarkers underlying neuropsychiatric, cognitive and behavioral manifestations in PD patients. These biomarkers could be used to improve the detection and recognition of neuropsychiatric manifestations of PD, such as depression and impulse control disorders. There is no data suggesting that modifying DBS parameters selectively improves these manifestations; hence, it would be premature to consider meaningfully integrating electrophysiological biomarkers of neuropsychiatric symptoms in multi‐input closed‐loop DBS algorithms for PD.

## 4 Discussion

STN LFP recordings from sensing‐enabled DBS systems offer a unique window into the electrophysiology of the STN and its association with PD symptoms. To date, biomarkers associated with non‐motor symptoms have been less explored, while biomarkers related to motor symptoms, especially in the beta and gamma frequency bands, are considered good electrophysiological biomarkers for closed‐loop DBS designs (Little et al. 2013; Little and Brown 2020; Oehrn et al. 2024; Velisar et al. 2019; Wang et al. 2023). Since non‐motor symptoms are often undertreated and can even overshadow motor symptoms in terms of their impact on quality of life (Jung et al. 2015; Schapira et al. 2017), identifying electrophysiological biomarkers reflecting non‐motor symptoms in PD is crucial, as it may improve their detection, monitoring and treatment. To do so, we reviewed the literature on STN LFP recordings in patients with non‐motor symptoms associated with PD.

Potential LFP biomarkers of non‐motor symptoms identified in the literature are summarized in Table 1. For pain and nociception, results from Parker et al. (2020) suggest that PD‐related pain is associated with a beta band power increase in the STN. Additionally, beta band power significantly decreases while STN‐DBS is activated (Parker et al. 2020). However, these same findings were not observed in another study by Belasen et al. (2016). Regarding sleep impairments, available results have established a significant increase in beta band power prior to sleep interruption (Anjum et al. 2024), suggesting that beta power could possibly be used as a biomarker for the management of sleep alterations in PD. Neuropsychiatric manifestations in PD are represented in the beta band (motor inhibition and reinforcement learning), but they are also represented in a wide range of other frequency bands (Ricciardi et al. 2023). For instance, depression is associated with a greater decrease in alpha activity in reaction to unpleasant stimuli (Huebl et al. 2011).

**TABLE 1:** Local field potential representation of physiological and pathological neuronal activity in the subthalamic nucleus in Parkinson's disease.

Frequency band	Physiological processes in PD	Non‐motor symptoms of PD
Delta (0–3 Hz)	Increase in N2/S2 stage sleep (Chen et al. 2019; Urrestarazu et al. 2009) Increase in NREM sleep (Thompson et al. 2018) Decrease in REM sleep (Chen et al. 2019)	
Theta (4–7 Hz)	Increase in N2/S2 stage sleep (Chen et al. 2019; Urrestarazu et al. 2009) Increase in NREM sleep (Thompson et al. 2018) Decrease in REM sleep (Chen et al. 2019) Increase during conflict processing and subjective valuation (Pearson et al. 2017; Ricciardi et al. 2023)	Increase associated with pathological gambling and impulse control disorders (Ricciardi et al. 2023; Rosa et al. 2013) Decrease associated with depressive symptomatology and negatively correlated with the severity of symptoms (Sun et al. 2021)
Alpha (8–12 Hz)	Decrease during sleep (Balachandar et al. 2024) Increase in N2/S2 stage sleep (Chen et al. 2019; Urrestarazu et al. 2009) Increase in NREM sleep (Thompson et al. 2018) Decrease in REM sleep (Chen et al. 2019) Involved in emotional processing (Ricciardi et al. 2023) Involved in reinforcement learning (Schroll et al. 2018) Increase following innocuous mechanical stimuli (Belasen et al. 2016)	Increase associated with depressive symptomatology and positively correlated with the severity of symptoms (Ricciardi et al. 2023; Sun et al. 2021) Reactivity diminution to pleasant stimuli and power decrease when presented with unpleasant stimuli for PD patients with depressive symptoms (Huebl et al. 2011)
Beta (13–30 Hz)	Decrease during sleep (Balachandar et al. 2024; van Rheede et al. 2022) Decrease during sleep stage S2 and S4 (Urrestarazu et al. 2009) Decrease during NREM sleep (Thompson et al. 2018) Decrease in N2 stage sleep (Chen et al. 2019) Decrease during N2 and N3 (Anjum et al. 2024) Increase in REM sleep (Urrestarazu et al. 2009) Increase prior to motor inhibition (Benis et al. 2014; Ricciardi et al. 2023) Involved in reinforcement learning (Schroll et al. 2018) Increase following mechanical painful stimuli (Parker et al. 2020)	Increase prior to sleep interruption (Anjum et al. 2024) Increase during REM sleep without atonia and positively correlated with the degree of atonia loss (Yin et al. 2024)
Gamma (35–100 Hz)	Decrease during NREM sleep (Thompson et al. 2018) Decrease in N2 stage sleep (Chen et al. 2019) Decrease with successful motor inhibition (Alegre et al. 2013)	Absence of expected reduction during motor inhibition for patients with impulse control disorders (Alegre et al. 2013)

Abbreviations: NREM: non‐rapid eye movement; PD: Parkinson's disease; REM: rapid eye movement.

### 4.1 From Neuronal Oscillations to Symptoms

To understand how disruptions in physiological neuronal oscillations may contribute to symptom manifestations in PD, it is essential to first explore the role of these oscillations in normal brain function, as various models have been proposed to explain this oscillatory activity (Buzsáki 2010; Fries 2015). Buzsáki's (2010) model proposes that oscillations coordinate groups of neurons, or cell assemblies. These assemblies fire synchronously, and oscillatory patterns bind them together, facilitating large‐scale organization across neural networks. Through this coordinated structure, the brain efficiently encodes complex information and adapts to environmental changes (Buzsáki 2010). Fries (2015) presented the model of communication through coherence, suggesting that phasic oscillations across brain regions enable efficient information exchange. When two regions oscillate coherently, they align their excitatory states, maximizing information transfer, a process essential for higher cognitive functions (Fries 2015). For patients with neurological disorders characterized by aberrant neuronal oscillations, these previously efficient, coordinated, and coherent processes, essential for managing the brain's complex network functions, might become disrupted, potentially explaining the emergence of these symptoms.

### 4.2 Limitations of the Studies Identified

First, the studies identified are promising but were realized on limited datasets. This hampers the ability to generalize findings and apply them more broadly in clinical settings. Furthermore, it is likely that the spectral representation of a specific symptom varies across patients, and large‐scale studies would be helpful to explore the specific response profiles observed. Second, most studies involved controlled experimental designs (e.g., task paradigms for motor inhibition or sensory testing for acute pain) conducted in controlled environments (e.g., during hospital visits or immediately after electrode implantation). While these studies can provide valuable mechanistic insights, they do not directly inform us about real‐time, at‐home fluctuations of symptoms, such as in chronic pain. Third, LFP recordings provide only a limited view of the complex landscape of brain activity. They presuppose a purely periodic activity in the STN, while many physiological states are encoded by aperiodic activity (Donoghue et al. 2020, 2022). The mechanisms behind the oscillations recorded in the STN are often difficult to explain, especially for less studied non‐motor manifestations. To gain a deeper understanding and a more complete picture of these mechanisms, multimodal correlative studies using both microelectrode recordings from single‐unit neurons and LFP would be beneficial, as they provide complementary information: single‐unit recordings capture precise output (spiking activity), while LFP reflects broader input activity and network dynamics (Nielsen et al. 2006). Fourth, although several biomarkers for motor and non‐motor symptoms have been identified, their clinical utility remains limited. Many studies show correlations at the group level, but these findings may not hold at the individual level, making their use potentially challenging in personalized treatment. Even when biomarkers demonstrate good predictive value, it is still unclear how we should use them in clinical settings. For instance, biomarkers could be used to monitor symptoms and assist clinicians in adjusting medication. However, there is no clear evidence that modifying DBS parameters can selectively target and improve non‐motor symptoms in a closed‐loop DBS fashion. Adjusting stimulation parameters to improve NMS may compromise the motor benefits typically seen with DBS. If many frequency bands are used to track many motor or non‐motor symptoms, the automated changes in stimulation parameters may lead to a suboptimal motor state or unwanted stimulation‐induced side effects.

### 4.3 Future Perspectives: Closed‐Loop DBS

Closed‐loop DBS systems operate autonomously by adapting stimulation parameters, such as amplitude or frequency, according to a specific input signal (Bouthour et al. 2019). For the treatment of motor symptoms, closed‐loop DBS offers an advantage over conventional open‐loop DBS (Oehrn et al. 2024). Notably, Little et al. (2013) tested a closed‐loop STN DBS system on eight patients with PD. The stimulation was triggered only when beta power reached a user‐defined threshold. The study showed a 29% improvement in motor symptoms and a 56% increase in battery life compared with conventional continuous DBS (Little et al. 2013). Oehrn et al. (2024) tested a different closed‐loop STN DBS system in four patients with PD. Using an additional subdural electrode to record from the frontal cortex, they described a potentially superior biomarker of motor state: the detection of a precise subharmonic frequency of the STN stimulation in the cortex, namely, a 65‐Hz power peak in response to a 130‐Hz stimulation (1:2 entrainment). Using these stimulation‐entrained gamma oscillations as a biomarker of motor state, they found that closed‐loop DBS reduced the awake “off” time by 16%, at the cost of delivering more total electrical energy compared to conventional continuous DBS in all patients (more time spent at higher stimulation amplitude during low dopaminergic periods) (Oehrn et al. 2024). As of now, there is no clear demonstration that parameter changes can selectively improve non‐motor symptoms in PD. Therefore, studying the electrophysiology of these symptoms in the STN mostly has fundamental scientific value, rather than direct clinical value in terms of closed‐loop DBS. Eventually, if a non‐motor manifestation is shown to be clinically predominant in a given patient, fluctuates throughout the day, responds to DBS parameter changes, and is clearly correlated with power changes in a LFP frequency band, then the electrophysiological biomarker of this symptom may be added to multi‐symptom closed‐loop DBS systems using complex multi‐input algorithms (Bouthour et al. 2019; Ricciardi et al. 2023). These algorithms would be able to (1) decode complex input signals (e.g., from different origins or frequency bands) and (2) personalize DBS parameters accordingly (Boutet et al. 2021; Little et al. 2013; Ricciardi et al. 2023; van Wijk et al. 2023; Wang et al. 2023). Inputs could be acquired not only from the STN but from other regions such as the sensorimotor cortex (Oehrn et al. 2024). Furthermore, a multi‐input approach acknowledges the complexity of PD, as it is unlikely that there is a simple one‐to‐one relationship between a particular frequency band and a specific symptom. Instead, each symptom is likely part of a complex electrophysiological network in the basal ganglia. Thus, symptoms are likely represented through various neurophysiological signals originating from multiple regions of the brain, or through different frequency bands from a single location like the STN (Maling and McIntyre 2016; Ricciardi et al. 2023). Additionally, a multi‐input approach could help account for interindividual differences in spectral representations within the STN. For example, it is estimated that 5% to 15% of patients with motor symptoms lack a peak in the beta band, meaning that relying solely on this frequency band to guide treatment might not be an optimal strategy (Crowell et al. 2012; Kuhn et al. 2006, 2009; Ozkurt et al. 2011; Ray et al. 2008; Stanslaski et al. 2024; Tinkhauser et al. 2017; Wang et al. 2016). This limitation could be addressed by using a weighted combination of spectral power across multiple frequency bands from the STN (Shah et al. 2023). However, for a real‐world, patient‐to‐patient perspective, and with the currently available technologies, implementing these multi‐input approaches may prove challenging, as training the complex models needed could become prohibitively time‐consuming. Exploring various parameter combinations to identify the optimal fit for the specific response being studied can be resource demanding. Notably, for conditions like treatment‐resistant depression, there is typically a latency period before the DBS system receives feedback on the effectiveness of prior parameter adjustments (Alagapan et al. 2023). A similar latency period might be expected for certain symptoms in PD. Given these challenges, it might be difficult to develop complex models capable of adjusting stimulation parameters within a reasonable amount of time. Finally, multi‐input algorithms could enable the simultaneous management of multiple motor and non‐motor symptoms, allowing the closed‐loop DBS system to determine an optimally weighted combination of parameters specific for each patient (Wang et al. 2023). However, adjusting stimulation parameters to address different symptoms may prove difficult, as it risks creating side effects and compromising the motor benefits achieved with DBS.

## 5 Conclusion

Research on DBS and hardware technology are progressing rapidly, with commercially available devices continuously improving, enabling cutting‐edge functionalities. To keep pace with these technological strides, ongoing studies are essential to deepen our understanding of the neuronal mechanisms underlying both motor and non‐motor symptoms in PD and to assess the impact of treatments on patients' quality of life.

## Author Contributions

**Marc‐Antoine Gobeil:** data curation (equal), formal analysis (equal), investigation (equal), methodology (supporting), visualization (equal), writing – original draft (equal), writing – review and editing (supporting). **Albert Guillemette:** conceptualization (lead), data curation (equal), formal analysis (equal), funding acquisition (supporting), investigation (equal), methodology (equal), project administration (equal), visualization (equal), writing – original draft (equal), writing – review and editing (lead). **Meziane Silhadi:** data curation (equal), writing – original draft (supporting). **Laurence Charbonneau:** data curation (supporting), writing – review and editing (supporting). **David Bergeron:** data curation (supporting), funding acquisition (supporting), methodology (supporting), project administration (supporting), supervision (supporting), writing – original draft (supporting), writing – review and editing (supporting). **Adan‐Ulises Dominguez‐Vargas:** data curation (supporting), writing – review and editing (supporting). **Numa Dancause:** supervision (supporting), writing – review and editing (supporting). **Nicolas Jodoin:** funding acquisition (supporting), writing – review and editing (supporting). **Elie Bou Assi:** data curation (supporting), writing – review and editing (supporting). **Florin Amzica:** data curation (supporting), writing – review and editing (supporting). **Sami Obaid:** data curation (supporting), supervision (supporting), writing – review and editing (supporting). **Marie‐Pierre Fournier‐Gosselin:** conceptualization (supporting), formal analysis (supporting), funding acquisition (lead), project administration (equal), resources (lead), supervision (lead), writing – original draft (supporting), writing – review and editing (supporting).

## Ethics Statement

The authors have nothing to report.

## Conflicts of Interest

The authors declare no conflicts of interest.

### Peer Review

The peer review history for this article is available at https://www.webofscience.com/api/gateway/wos/peer‐review/10.1111/ejn.70046.

## Supporting information


**Data S1.** Supporting information

## Data Availability

The authors have nothing to report.
