# Supplementary material for: Local Field Potential Biomarkers of Non‐Motor Symptoms in Parkinson's Disease: Insights From the Subthalamic Nucleus in Deep Brain Stimulation
Source: Eur J Neurosci. 2025 Mar 4;61(5):e70046. doi: 10.1111/ejn.70046 (PMC11880747; doi:10.1111/ejn.70046)
Supplement: Supplementary file 1 — Data S1. Supporting information [file EJN-61-0-s001.pdf]

Scale for the Assessment of Narrative Review Articles – SANRA

Please rate the quality of the narrative review article in question, using categories 0–2 on the following scale. For each aspect of quality, please choose the option which best fits your evaluation, using categories 0 and 2 freely to imply general low and high quality. These are not intended to imply the worst or best imaginable quality.

1) Justification of the article's importance for the readership

- The importance is not justified. — 0
- The importance is alluded to, but not explicitly justified. — 1
- The importance is explicitly justified. — 2

☐

2) Statement of concrete aims or formulation of questions

- No aims or questions are formulated. — 0
- Aims are formulated generally but not concretely or in terms of clear questions. — 1
- One or more concrete aims or questions are formulated. — 2

☐

3) Description of the literature search

- The search strategy is not presented. — 0
- The literature search is described briefly. — 1
- The literature search is described in detail, including search terms and inclusion criteria. — 2

☐

4) Referencing

- Key statements are not supported by references. — 0
- The referencing of key statements is inconsistent. — 1
- Key statements are supported by references. — 2

☐

5) Scientific reasoning

(e.g., incorporation of appropriate evidence, such as RCTs in clinical medicine)

- The article's point is not based on appropriate arguments. — 0
- Appropriate evidence is introduced selectively. — 1
- Appropriate evidence is generally present. — 2

☐

6) Appropriate presentation of data

(e.g., absolute vs relative risk; effect sizes without confidence intervals)

- Data are presented inadequately. — 0
- Data are often not presented in the most appropriate way. — 1
- Relevant outcome data are generally presented appropriately. — 2

☐

Sumscore ☐

SANRA – explanations and instructions

This scale is intended to help editors assess the quality of a narrative review article based on formal criteria accessible to the reader. It cannot cover other elements of editorial decision making such as degree of originality, topicality, conflicts of interest or the plausibility, correctness or completeness of the content itself. SANRA is an instrument for editors, authors, and reviewers evaluating individual manuscripts. It may also help editors to document average manuscript quality within their journal and researchers to document the manuscript quality, for example in peer review research. Using only three scoring options, 0, 1 and 2, SANRA is intended to provide a swift and pragmatic sum score for quality, for everyday use with real manuscripts, in a field where established quality standards have previously been lacking. It is not designed as an exact measurement of the quality of all theoretically possible manuscripts. For this reason, the extreme values (0 and 2) should be used relatively freely and not reserved only for perfect or hopeless articles.

We recommend that users test-rate a few manuscripts to familiarize themselves with the scale, before using it on the intended group of manuscripts. Ratings should assess the totality of a manuscript, including the abstract. The following comments clarify how each question is designed to be used.

Item 1 – Justification of the article's importance for the readership

Justification of importance for the readership must be seen in the context of each journal's readership.
Consider how well the manuscript outlines the clinical problem and highlights unanswered questions or evidence gaps – thoroughly (2), superficially (1), or not at all (0).

Item 2 – Statement of concrete/specific aims or formulation of questions

A good paper will propose one or more specific aims or questions which will be dealt with or topics which will be reviewed.
Please rate whether this has been done thoroughly and clearly (2), vaguely or unclearly (1), or not at all (0).

Item 3 – Description of the literature search

A convincing narrative review will be transparent about the sources of information on which the text is based. Please rate the degree to which you think this has been achieved. To achieve a rating of 2, it is not necessary to describe the literature search in as much detail as for a systematic review (searching multiple databases, including exact descriptions of search history, flowcharts, etc.), but it is necessary to specify search terms, and the types of literature included. A manuscript which only refers briefly to its literature search would score 1, while one not mentioning its methods would score 0.

Item 4 – Referencing

No manuscript references all statements. However, those that are essential for the arguments of the manuscript – "key statements" – should be backed by references in all or almost all cases. Exceptions could reasonably be made for rating purposes where a key statement has uncontroversial face-validity, such as "Diabetes is among the commonest causes of chronic morbidity worldwide."
Please rate the completeness of referencing: for most or all relevant key statements (2), inconsistently (1), sporadically (0).

Item 5 – Scientific reasoning

The item describes the quality of the scientific point made. A convincing narrative review presents evidence for key arguments.
It should mention study design (randomized controlled trial, qualitative study, etc), and where available, levels of evidence.
Please rate whether you feel this has been done thoroughly (2), superficially (1), or hardly at all (0). Unlike item 6, which is concerned with the selection and presentation of concrete outcome data, this item relates to the use of evidence and of types of evidence in the manuscript's arguments.

Item 6 – Appropriate presentation of data:

This item describes the correct presentation of data central to the article's argument. Which data are considered relevant varies from field to field. In some areas relevant data would be absolute rather than relative risks or clinical versus surrogate or intermediate endpoints. These outcomes must be presented correctly. For example, it is appropriate that effect sizes are accompanied by confidence intervals. Please rate how far the paper achieves this – thoroughgoingly (2), partially (1), or hardly at all (0). Unlike item 5, which relates to the use of evidence and of types of evidence in the manuscript's arguments, this item is concerned with the selection and presentation of concrete outcome data.

Reference

Baethge C, Goldbeck-Wood S, Mertens S: SANRA—a scale for the quality assessment of narrative review articles. Research Integrity and Peer Review (2019) 4:5
https://doi.org/10.1186/s41073-019-0064-8

PRISMA 2020 Checklist

Section and Topic	Item #	Checklist item	Location where item is reported
**TITLE**			
Title	1	Identify the report as a systematic review.	
**ABSTRACT**			
Abstract	2	See the PRISMA 2020 for Abstracts checklist.	
**INTRODUCTION**			
Rationale	3	Describe the rationale for the review in the context of existing knowledge.	
Objectives	4	Provide an explicit statement of the objective(s) or question(s) the review addresses.	
**METHODS**			
Eligibility criteria	5	Specify the inclusion and exclusion criteria for the review and how studies were grouped for the syntheses.	
Information sources	6	Specify all databases, registers, websites, organisations, reference lists and other sources searched or consulted to identify studies. Specify the date when each source was last searched or consulted.	
Search strategy	7	Present the full search strategies for all databases, registers and websites, including any filters and limits used.	
Selection process	8	Specify the methods used to decide whether a study met the inclusion criteria of the review, including how many reviewers screened each record and each report retrieved, whether they worked independently, and if applicable, details of automation tools used in the process.	
Data collection process	9	Specify the methods used to collect data from reports, including how many reviewers collected data from each report, whether they worked independently, any processes for obtaining or confirming data from study investigators, and if applicable, details of automation tools used in the process.	
Data items	10a	List and define all outcomes for which data were sought. Specify whether all results that were compatible with each outcome domain in each study were sought (e.g. for all measures, time points, analyses), and if not, the methods used to decide which results to collect.	
	10b	List and define all other variables for which data were sought (e.g. participant and intervention characteristics, funding sources). Describe any assumptions made about any missing or unclear information.	
Study risk of bias assessment	11	Specify the methods used to assess risk of bias in the included studies, including details of the tool(s) used, how many reviewers assessed each study and whether they worked independently, and if applicable, details of automation tools used in the process.	
Effect measures	12	Specify for each outcome the effect measure(s) (e.g. risk ratio, mean difference) used in the synthesis or presentation of results.	
Synthesis methods	13a	Describe the processes used to decide which studies were eligible for each synthesis (e.g. tabulating the study intervention characteristics and comparing against the planned groups for each synthesis (item #5)).	
	13b	Describe any methods required to prepare the data for presentation or synthesis, such as handling of missing summary statistics, or data conversions.	
	13c	Describe any methods used to tabulate or visually display results of individual studies and syntheses.	
	13d	Describe any methods used to synthesize results and provide a rationale for the choice(s). If meta-analysis was performed, describe the model(s), method(s) to identify the presence and extent of statistical heterogeneity, and software package(s) used.	
	13e	Describe any methods used to explore possible causes of heterogeneity among study results (e.g. subgroup analysis, meta-regression).	
	13f	Describe any sensitivity analyses conducted to assess robustness of the synthesized results.	
Reporting bias assessment	14	Describe any methods used to assess risk of bias due to missing results in a synthesis (arising from reporting biases).	
Certainty assessment	15	Describe any methods used to assess certainty (or confidence) in the body of evidence for an outcome.	

PRISMA 2020 Checklist

Section and Topic	Item #	Checklist item	Location where item is reported
**RESULTS**			
Study selection	16a	Describe the results of the search and selection process, from the number of records identified in the search to the number of studies included in the review, ideally using a flow diagram.	
	16b	Cite studies that might appear to meet the inclusion criteria, but which were excluded, and explain why they were excluded.	
Study characteristics	17	Cite each included study and present its characteristics.	
Risk of bias in studies	18	Present assessments of risk of bias for each included study.	
Results of individual studies	19	For all outcomes, present, for each study: (a) summary statistics for each group (where appropriate) and (b) an effect estimate and its precision (e.g. confidence/credible interval), ideally using structured tables or plots.	
Results of syntheses	20a	For each synthesis, briefly summarise the characteristics and risk of bias among contributing studies.	
	20b	Present results of all statistical syntheses conducted. If meta-analysis was done, present for each the summary estimate and its precision (e.g. confidence/credible interval) and measures of statistical heterogeneity. If comparing groups, describe the direction of the effect.	
	20c	Present results of all investigations of possible causes of heterogeneity among study results.	
	20d	Present results of all sensitivity analyses conducted to assess the robustness of the synthesized results.	
Reporting biases	21	Present assessments of risk of bias due to missing results (arising from reporting biases) for each synthesis assessed.	
Certainty of evidence	22	Present assessments of certainty (or confidence) in the body of evidence for each outcome assessed.	
**DISCUSSION**			
Discussion	23a	Provide a general interpretation of the results in the context of other evidence.	
	23b	Discuss any limitations of the evidence included in the review.	
	23c	Discuss any limitations of the review processes used.	
	23d	Discuss implications of the results for practice, policy, and future research.	
**OTHER INFORMATION**			
Registration and protocol	24a	Provide registration information for the review, including register name and registration number, or state that the review was not registered.	
	24b	Indicate where the review protocol can be accessed, or state that a protocol was not prepared.	
	24c	Describe and explain any amendments to information provided at registration or in the protocol.	
Support	25	Describe sources of financial or non-financial support for the review, and the role of the funders or sponsors in the review.	
Competing interests	26	Declare any competing interests of review authors.	
Availability of data, code and other materials	27	Report which of the following are publicly available and where they can be found: template data collection forms; data extracted from included studies; data used for all analyses; analytic code; any other materials used in the review.	

From: Page MJ, McKenzie JE, Bossuyt PM, Boutron I, Hoffmann TC, Mulrow CD, et al. The PRISMA 2020 statement: an updated guideline for reporting systematic reviews. BMJ 2021;372:n71. doi: 10.1136/bmj.n71
